# MRMS-CNNFormer: A Novel Framework for Predicting the Biochemical Recurrence of Prostate Cancer on Multi-Sequence MRI

**DOI:** 10.3390/bioengineering12050538

**Published:** 2025-05-16

**Authors:** Tao Lian, Mengting Zhou, Yangyang Shao, Xiaqing Chen, Yinghua Zhao, Qianjin Feng

**Affiliations:** 1 School of Biomedical Engineering, Southern Medical University, Guangzhou 510515, China; taolian92@gmail.com; 2 Department of Radiology, The Third Affiliated Hospital, Southern Medical University, Guangzhou 510630, China; zhouxiaomi@smu.edu.cn (M.Z.); shaoyy58588@163.com (Y.S.); 3 Department of Radiology, The Second Affiliated Hospital, School of Medicine, The Chinese University of Hong Kong, Shenzhen 518172, China; 4 Department of Radiology, The Second Clinical Medical College of Guangzhou University of Chinese Medicine, Guangdong Provincial Hospital of Traditional Chinese Medicine, Guangzhou 510210, China; chenxiaqing0804@163.com; 5 Guangdong Provincial Key Laboratory of Medical Image Processing, Southern Medical University, Guangzhou 510515, China; 6 Guangdong Province Engineering Laboratory for Medical Imaging and Diagnostic Technology, Southern Medical University, Guangzhou 510515, China

**Keywords:** prostate cancer, biochemical recurrence, magnetic resonance imaging, deep learning, preoperative prognostic prediction

## Abstract

Accurate preoperative prediction of biochemical recurrence (BCR) in prostate cancer (PCa) is essential for treatment optimization, and demands an explicit focus on tumor microenvironment (TME). To address this, we developed MRMS-CNNFormer, an innovative framework integrating 2D **m**ulti-**r**egion (intratumoral, peritumoral, and periprostatic) and **m**ulti-**s**equence magnetic resonance imaging (MRI) images (T2-weighted imaging with fat suppression (T2WI-FS) and diffusion-weighted imaging (DWI)) with clinical characteristics. The framework utilizes a **CNN**-based encoder for imaging feature extraction, followed by a trans**former**-based encoder for multi-modal feature integration, and ultimately employs a fully connected (FC) layer for final BCR prediction. In this multi-center study (46 BCR-positive cases, 186 BCR-negative cases), patients from centers A and B were allocated to training (*n* = 146) and validation (*n* = 36) sets, while center C patients (*n* = 50) formed the external test set. The multi-region MRI-based model demonstrated superior performance (AUC, 0.825; 95% CI, 0.808–0.852) compared to single-region models. The integration of clinical data further enhanced the model’s predictive capability (AUC 0.835; 95% CI, 0.818–0.869), significantly outperforming the clinical model alone (AUC 0.612; 95% CI, 0.574–0.646). MRMS-CNNFormer provides a robust, non-invasive approach for BCR prediction, offering valuable insights for personalized treatment planning and clinical decision making in PCa management.

## 1. Introduction

Prostate cancer (PCa) remains the second most diagnosed cancer in men and a leading cause of cancer-related mortality worldwide [1]. Radical prostatectomy (RP) serves as the primary treatment for clinically localized PCa; however, approximately 20–40% of patients develop biochemical recurrence (BCR), characterized by two consecutive prostate-specific antigen (PSA) elevations above 0.2 ng/mL following surgery [2,3,4]. BCR represents a critical clinical endpoint, as it significantly correlates with increased risks of disease progression, metastasis, and mortality [5]. Current BCR risk assessment methods include postoperative PSA kinetics monitoring, risk assessment systems like CAPRA, and genomic biomarker testing [6,7,8,9]. Despite their clinical utility, these approaches present several limitations: invasive sampling requirements, delayed intervention possibilities, variable predictive accuracy, and substantial costs. These limitations underscore the pressing need for developing cost-effective and non-invasive preoperative BCR prediction strategies.

Radiologists utilize diverse magnetic resonance imaging (MRI) features for BCR prediction in PCa patients [10]. Structural features, such as tumor volume, extracapsular extension (ECE), and seminal vesicle invasion (SVI), serve as primary diagnostic indicators [11]. In quantitative assessment, apparent diffusion coefficient (ADC) values and tumor margin irregularity emerge as significant predictive markers [12]. Moreover, standardized reporting systems, particularly PI-RADS v2 scores (4–5), demonstrate strong correlation with elevated BCR risk following treatment [13]. Despite their clinical utility, these imaging biomarkers face substantial challenges in reliability: inter-scanner variability, image-quality inconsistencies, and observer-dependent interpretation. These technical and operational limitations necessitate the development of robust quantitative methods for extracting reproducible and clinically meaningful information from MRI sequences, thereby enhancing BCR prediction accuracy.

MRI has emerged as a promising tool for predicting BCR in PCa through its advanced quantitative imaging capabilities and artificial intelligence-based analysis. Recent studies [14,15] have highlighted the ability of medical imaging techniques to detect both local and metastatic recurrence with significantly higher sensitivity compared to conventional methods. The integration of multiparametric MRI (mpMRI) features with clinical parameters has further enhanced predictive accuracy, particularly through deep learning approaches, has further enhanced predictive accuracy [14,16,17]. Several pioneering studies have validated the efficacy of MRI-based BCR prediction through different methodological approaches. In conventional imaging analysis, Park et al. [18] identified tumor volume and ADC values as significant predictors using 3T mpMRI, demonstrating higher BCR rates in patients with larger tumor volumes and lower ADC values. Advancing to radiomics analysis, researchers developed a comprehensive model incorporating 1536 features extracted from T1, T2, and DWI sequences, achieving an Area Under the Curve (AUC) of 0.73 in their test cohort [19]. Similarly, another study combining mpMRI features with clinical parameters achieved an AUC of 0.77 for BCR risk assessment [15]. Most notably, Lee et al. [14] established the superiority of deep learning approaches by demonstrating enhanced discriminative performance of deep learning-derived features over conventional features in predicting BCR-free survival. These progressive advances in MRI analysis techniques collectively underscore MRI’s potential as a non-invasive and highly effective tool for BCR prediction in PCa patients.

Conventional BCR prediction models predominantly focus on tumor-centric features, overlooking crucial prognostic information embedded within the TME and its dynamic interactions with adjacent tissues. Recent studies [20] have demonstrated that both peritumoral and intratumoral regions harbor valuable predictive features for BCR, with peritumoral tissue characteristics offering unique insights into tumor progression and metastatic potential. Moreover, developing advanced multi-sequence MRI integration methods rather than relying solely on single-sequence analysis presents an opportunity to more comprehensively capture complementary information across different sequences for improved prediction performance.

To this end, we propose a hybrid approach that combines convolutional neural networks (CNN) for feature extraction with transformer-based architectures for feature fusion. While CNN excels at capturing local spatial patterns and hierarchical features from medical images, transformer [21,22,23,24,25] inherently excels at modeling global dependencies through their self-attention mechanism and effectively handling heterogeneous inputs. This complementary architectural design enables the comprehensive feature integration of multimodal data: imaging features (from multiple MRI sequences and distinct anatomical regions) and clinical data. Building on these strengths, we develop the MRMS-CNNFormer framework. The main contributions of our work could be summarized as follows:

(1) We propose MRMS-CNNFormer, a novel analysis framework that integrates multi-region MRI (intratumoral, peritumoral, and periprostatic regions) with multi-sequence imaging (T2-weighted imaging with fat suppression (T2WI-FS) and diffusion-weighted imaging (DWI)) for the comprehensive characterization of the tumor microenvironment, addressing the limitations of conventional tumor-centric approaches.
(2) We develop a hierarchical feature fusion architecture that combines CNN-based encoder for regional feature extraction with a transformer-based encoder for the cross-modal integration of imaging and clinical data, effectively capturing spatial relationships and complementary information across different anatomical regions, imaging sequences, and clinicopathological variables for enhanced prognostic assessment.
(3) Experiments are conducted on a multi-center dataset, demonstrating that MRMS-CNNFormer outperforms both single-region models (AUC 0.835 vs. 0.658–0.803) and clinical-only models (AUC 0.835 vs. 0.612) in predicting BCR on the external testing dataset, providing a robust, non-invasive tool for personalized treatment planning in prostate cancer management.

## 2. Method

**Figure 1:**
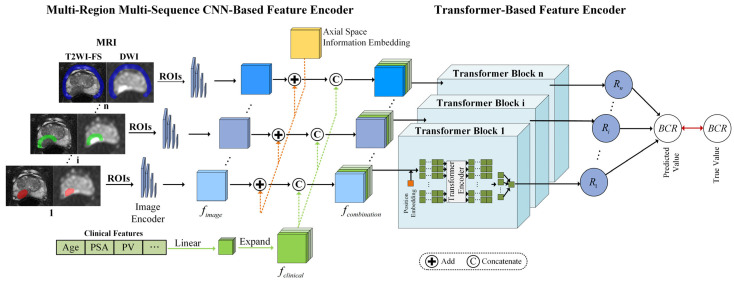
The framework of the proposed MRMS-CNNFormer for predicting the BCR using MRI sequences. The intratumoral, peritumoral, and periprostatic regions are marked by red, green, and blue, respectively.

In this section, we present a comprehensive description of our proposed MRMS-CNNFormer architecture. As illustrated in Figure 1, the MRMS-CNNFormer framework comprises two principal components. The first component implements a multi-region, multi-sequence CNN-based feature encoder that systematically extracts discriminative features across diverse spatial regions. The second component utilizes a transformer-based feature encoder to effectively fuse multi-modal features from imaging and clinical data. Finally, the integrated features are fed into an FC layer to generate precise BCR prediction values. The architectural details of each component are elaborated in the following subsections.

### 2.1. Multi-Region Multi-Sequence CNN-Based Feature Encoder

#### 2.1.1. MRI Slice Feature Extraction

To enhance computational efficiency, our approach begins by converting 3D MRI volumes into sequential 2D axial slices. For each slice, we employ mask-based segmentation to isolate three distinct ROIs. The preprocessed T2WI-FS and DWI sequences are then combined to create dual-channel inputs, preserving complementary diagnostic information from the two sequences. Feature extraction is performed using ResNet18 [26] as the backbone architecture, leveraging its efficient residual structure to generate comprehensive representations of the multi-sequence MRI slices. Since all slices undergo identical processing, we will focus on describing the propagation pathway of a single combined slice through our model.

During the convolutional processing stage, the stacked MRI image $I\in {\mathbb{R}}^{W\times H\times 2}$ serves as the network input. The feature representation extracted through the convolutional network is denoted as $f_{image}=SliceEncoder(I)\in {\mathbb{R}}^{W_{I}\times H_{I}\times C_{I}}$, where $W_{I}$, $H_{I}$, and $C_{I}$ represent the width, height, and channel dimensions of the resulting feature tensor, respectively.

#### 2.1.2. Axial Space Information Embedding

The use of a 2D axial dataset inevitably results in the loss of inherent spatial context along the axial dimension. Additionally, standardizing slice alignment across patients presents challenges due to anatomical variations and differences in slice quantities. To address these limitations, we incorporate axial spatial information embedding for each 2D slice. This embedding, represented as $AS\in {\mathbb{R}}^{W_{I}\times H_{I}}$, is randomly initialized and optimized during the training process. This strategy prevents the model from relying solely on relative slice positions, enabling a more effective alignment of slices across different patients.

The axial spatial feature tensor is extended along the channel dimension to match the dimensions of the image feature. The expanded axial spatial position feature is denoted as $f_{as}\in {\mathbb{R}}^{W_{I}\times H_{I}\times C_{I}}$. Subsequently, corresponding elements at identical spatial positions in $f_{as}$ and $f_{image}$ are directly combined through element-wise addition to create a new image feature incorporating axial spatial location information. The resulting enhanced image feature is expressed as $f_{ASI}=f_{as}+f_{image}$.

#### 2.1.3. Clinical Feature Construction

Clinical data comprises two primary categories: numerical variables and categorical variables. Categorical information (such as ISUP grade) undergoes numerical encoding, while numerical data (such as patient age) are normalized to the $\left[0,\;1\right]$ range through min–max scaling. Following the integration of axial spatial information into the image features, the processed clinical data are incorporated with the image features. Since the dimensionality of clinical data differs from that of image features, alignment is necessary. To resolve this dimensional mismatch, clinical features are expanded to match the dimensions of the image features, facilitating the comprehensive integration of clinical and imaging information.

**Figure 2:**
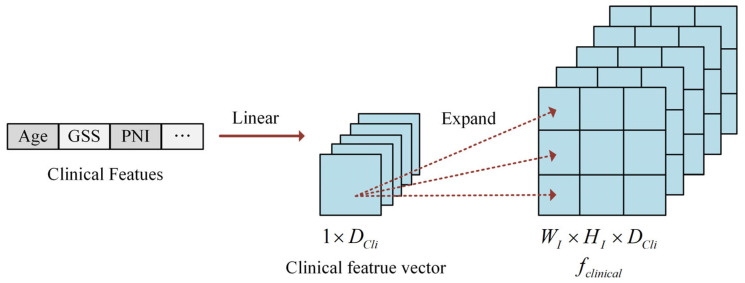
The illustration of clinical feature construction.

The two categories of clinical data are combined to form a $1\times D_{Cli}$ vector, as illustrated in Figure 2. To explore the relationships between individual clinical parameters and imaging characteristics, the original clinical feature vector undergoes linear transformation and dimensional expansion to align with the image-feature dimensions. Specifically, each element in the clinical feature vector is replicated to create a matrix matching the width $W_{I}$ and height $H_{I}$ of the slice image. This process generates the expanded clinical feature representation, denoted as $f_{clinical}\in {\mathbb{R}}^{W_{I}\times H_{I}\times D_{Cli}}$.

### 2.2. Transformer-Based Feature Encoder

In the transformer-based feature encoder module, the clinical features $f_{clinical}$ are concatenated with the spatially enhanced image features $f_{ASI}$ along the channel dimension to create a comprehensive multi-modal representation $f_{combination}\in {\mathbb{R}}^{W_{I}\times H_{I}\times (C_{I}+D_{Cli})}$. Within the transformer block, since the transformer encoder requires sequential input, each $f_{combination}$ is reshaped to conform to the input specifications of the transformer encoder. Specifically, each voxel of the combination feature along the channel dimension was treated as a sequence of combination features (SCF). To further enhance SCF learning, positional embeddings are incorporated into the SCFs, enabling the transformer encoder to effectively capture the SCF order. The component vectors along the channel dimension C are represented as $\left[c_{1},\;c_{2},\;\ldots ,\;c_{S}\right]$, generating S SCFs, where $S=W_{I}\times H_{I}$.

The transformer encoder processes the sequence of S SCFs and produces an output vector $V\in {\mathbb{R}}^{S*C}$, which represents C predicted values. The C values derived from the fusion features of the i−th slice are then transformed through an FC layer to generate an element $R_{i}$ of the recurrence vector R. This process can be mathematically formulated as:

(1) $$R_{i}=W_{FC}\times TransformerFeatureEncoder(f_{combination})+B_{FC}$$

where $W_{FC}$ and $B_{FC}$ represent the weight matrix and bias vector of the FC layer, respectively.

For the recurrence vector R, we consider that each patient’s 3D MRI examination is divided into *N* slices. Each slice contributes partial information about the patient’s recurrence status, reflecting distinct spatial and physiological characteristics that influence their relative importance in predicting the final recurrence probability. Based on this principle, we construct a predicted recurrence vector R, denoted as $R=\left[r_{1},r_{2},\ldots ,r_{n}\right]$, which comprises the predicted recurrence values generated from each slice of a patient through the transformer block.

Finally, an FC layer is employed to derive the overall predicted recurrence outcome for the patient. The entire model is optimized by comparing this predicted value with the ground-truth annotation. The prediction loss function is expressed as:

(2) $$Loss=-\frac{1}{N}\sum_{i=1}^{N}\left[y_{i}\log(\overset{\wedge }{y_{i}})+(1-y_{i})\log(1-\overset{\wedge }{y_{i}})\right]$$

where $\overset{\wedge }{y_{i}}$ represents the predicted recurrent probability, $y_{i}$ denotes the ground-truth label, and Loss signifies the binary cross-entropy loss function.

## 3. Materials and Experimental Configurations

### 3.1. Patient Cohorts

This multicenter retrospective study was conducted at three tertiary medical centers in South China (Center A, Center B and Center C) between January 2013 and December 2020. The study protocol was approved by the institutional review boards of all three centers. All institutional review boards independently reviewed and approved the research methodology and data collection procedures. Given the retrospective nature of this study, written informed consent was waived by all three institutional review boards.

Patient selection followed a systematic two-step screening process. Initially, eligible participants were required to meet all the following inclusion criteria: (1) biopsy-confirmed primary PCa; (2) the completion of preoperative mpMRI examinations following standardized protocols; and (3) documented BCR, defined as two consecutive postoperative PSA measurements ≥0.2 ng/mL at least two weeks apart. Subsequently, patients were excluded if they met any of the following conditions: (1) receipt of additional therapeutic interventions beyond RP, including neoadjuvant hormonal therapy, radiotherapy, or chemotherapy; (2) incomplete clinical or follow-up data, specifically missing PSA measurements, unclear pathological staging documentation; (3) evidence of distant metastases on preoperative imaging; or (4) inadequate MRI quality, characterized by the absence of T2WI-FS or DWI sequences, or the presence of significant motion artifacts compromising diagnostic accuracy.

Clinical data, including demographic characteristics, preoperative PSA levels, biopsy Gleason scores, and pathological outcomes, were systematically collected from electronic medical records using standardized data extraction forms.

All MRI examinations were performed using either 1.5T (Philips Achieva and Multiva, Philips Healthcare, Best, The Netherlands) or 3.0T (Philips Achieva and Ingenia, Philips Healthcare, Best, The Netherlands) scanners equipped with either a pelvic surface phased array coil (PPAC) or an endorectal coil (ERC) across the three centers. The imaging protocol focused on T2WI-FS and DWI sequences. The imaging parameters for T2WI-FS and DWI sequences were as follows: repetition time/echo time (TR/TE) ranges of 1680–3522 ms/80–100 ms and 2000–6000/61–90 ms, respectively; a slice thickness of 3–4 mm with minimal gaps (0–1 mm); and with the field of view (FOV) ranging from 180 × 180 to 410 × 318 mm^2^. DWI was acquired using a specific b-value for each patient, with b-values varying across different centers and even among patients within the same center, and the b-values utilized throughout the entire dataset included 800, 1000, 1200, 1500, and 2000 s/mm^2^. Total acquisition time ranged from 1.5 to 5 min per sequence. All images were independently evaluated by two radiologists with more than 5 years of experience in prostate imaging, with any discrepancies resolved through consensus review.

A total of 232 patients were enrolled in this multi-center study. Using stratified randomization, patients from Centers A and B were allocated to the training (80%, *n* = 146) set and validation (20%, *n* = 36) set based on 5-fold cross-validation repeated 20 times. To evaluate the model’s generalizability, all patients from Center C (*n* = 50) were assigned to an independent external test set, ensuring the rigorous assessment of the model’s performance across diverse clinical settings. All images were preprocessed using the z-score normalization method.

### 3.2. MRI Segmentation

Original MRI data were retrieved from the hospital’s Picture Archiving and Communication System (PACS). Image analysis was performed independently by two board-certified radiologists (S.Y.Y. and Z.M.T.), with 3 and 5 years of experience in prostate MRI interpretation, respectively. The radiologists, blinded to clinical information, simultaneously segmented both tumor regions and prostate glands on T2WI-FS and DWI sequences. Subsequently, two standardized regions were established through morphological operations for multi-regional MRI analysis. The first region, referred to as the PTR, was generated by expanding the tumor boundary ROI by 5 mm while excluding extra-prostatic areas. The second region, termed the PPR, was created through 5 mm morphological dilation of the prostate contour ROI, with adjacent organs carefully excluded [27]. To ensure quality control, any discordant delineations were resolved through arbitration by a senior radiologist (Y.Q.), who had 7 years of experience in genitourinary imaging. The reliability of the segmentations was rigorously assessed through both inter- and intra-observer evaluations using the Dice Similarity Coefficient (DSC), a widely accepted metric for quantifying segmentation accuracy and consistency in medical image analysis [28].

### 3.3. Comparison of Results with Different Region Inputs and Clinical Data

We conducted a comprehensive evaluation of the proposed MRMS-CNNFormer model through systematic architectural comparisons. The model’s performance was assessed using three distinct MRI-derived ROIs: ITR, PTR, and PPR. To explore the potential synergistic effects, we developed two enhanced architectures: (1) a combined model that integrates features from all three ROIs via multi-regional fusion, and (2) an integrated model that incorporates both the combined imaging features and clinically validated prognostic factors.

Specifically, the combined model utilizes complementary information from ITR, PTR, and PPR through a multimodal fusion architecture, while the integrated model further improves predictive capability by incorporating key clinicopathological variables known to influence biochemical recurrence. This hierarchical approach allows for the systematic evaluation of the contribution from each information source to the overall prognostic performance.

### 3.4. Experimental Configuration

For the backbone CNN architecture, we utilized ResNet18 pre-trained on ImageNet to leverage transfer learning benefits. The fine-tuning strategy included freezing the first two layers and fine-tuning the remaining layers. To accommodate our dual-channel input (T2WI-FS and DWI), we modified the first convolutional layer by averaging the RGB channel weights of the pre-trained model and duplicating them to form a two-channel input layer while preserving the learned feature-extraction capabilities. The transformer encoder comprised 4 layers, 8 attention heads, and a 256-dimensional hidden representation, initialized with Xavier uniform distribution to facilitate effective gradient propagation during training.

The framework was implemented in PyTorch (version 1.9.0) and trained on an NVIDIA TESLA V100 GPU (NVIDIA Corporation, Santa Clara, CA, USA) with 32 GB memory. We utilized the AdamW optimizer with an initial learning rate of 0.0001. The learning rate was adjusted using a cosine annealing schedule. A dropout rate of 0.1 was applied after each transformer layer. The model was trained with a batch size of 16 for 300 epochs with early stopping.

Specifically, the AdamW optimizer prevents overfitting by implementing weight decay directly in the parameter update step, independent of adaptive learning rates, effectively reducing model complexity while maintaining optimization efficiency. The early stopping mechanism automatically halts the training process if validation performance shows no improvement for 10 consecutive epochs. The batch normalization layers integrated into the CNN architecture stabilize training dynamics and the dropout included in the transformer encoder constrains model complexity. Extensive data augmentation techniques—such as random rotation, flipping, brightness, and contrast adjustments—were employed to artificially expand the training dataset and improve generalization. Moreover, to ensure robustness to data division, we conducted 5-fold cross-validation repeated 20 times and reported the average values and 95% confidence intervals (CIs).

### 3.5. Statistical Analysis

All statistical analyses were conducted using R software (version 4.2.3) and SPSS (version 24.0). Continuous variables were summarized as mean (standard deviation) or median (interquartile range), while categorical variables were presented as number (percentage). Between-group differences in statistical and clinicopathological characteristics were assessed using the two-sample *t*-test or Wilcoxon rank-sum test for continuous variables, and the Chi-square test or Fisher’s exact test for categorical variables. The continuous variables were tested for normality prior to statistical analysis. We employed the Shapiro–Wilk test to assess data distribution, with *p* values < 0.05 considered as a significant deviation from normality. The normally distributed variables were analyzed using an independent samples *t*-test, while non-normally distributed variables were compared using the Mann–Whitney U test. Model performance was evaluated through ROC curve analysis, incorporating metrics such as AUC, sensitivity, specificity, accuracy, and F1-score. The DeLong test was used to compare ROC curves of different predictive models and evaluate the statistical significance of AUC differences. For all hypothesis tests, two-sided *p* values < 0.05 were considered statistically significant. The performance metrics were calculated using the following formulas:

(3) $$\mathrm{Sensitivity}=\frac{TP}{TP+FN}$$

(4) $$\mathrm{Specificity}=\frac{TN}{TN+FP}$$

(5) $$\mathrm{Accuracy}=\frac{TP+TN}{TP+TN+FP+FN}$$

(6) $$F1-\mathrm{score}=\frac{2\times TP}{2\times TP+FP+FN}$$

where *TP* is true positive, *TN* is true negative, *FP* is false positive, and *FN* is false negative.

## 4. Results

### 4.1. Patients

This retrospective study encompassed 232 patients, with 46 cases of BCR and 186 cases without BCR. The study population was divided into internal and external datasets. The internal dataset comprised 182 patients with a median age of 71.0 years (interquartile range: 65.0–76.0 years), among whom 38 patients developed BCR. The external dataset included 50 patients with a median age of 68.0 years (interquartile range: 62.8–73.0 years), with 8 patients experiencing BCR. Comparative analysis revealed statistically significant differences (*p* < 0.05) between the internal and external datasets across multiple clinical parameters, including age, Gleason score sum, ISUP grade, clinical stage, pathological stage, and CAPRA score. Regarding methodological validation, ROI delineation demonstrated high reliability, with a DSC of 0.92 and 0.95 for inter-observer and intra-observer assessments, respectively. Further details are illustrated in Table 1.

### 4.2. Performance of the Multi-Regional MRI-Based Models

The comparative analysis of MRI-based models for BCR prediction, as detailed in Table 2, demonstrated that the combined multi-region model consistently outperformed single-region models across all datasets. In the training set (*n* = 146), the combined model achieved superior performance metrics, with an AUC of 0.835 (95% CI: 0.812–0.855), a sensitivity of 0.788 (95% CI: 0.759–0.806), a specificity of 0.859 (95% CI: 0.834–0.887), an accuracy of 0.852 (95% CI: 0.817–0.866), and an F1-score of 0.675 (95% CI: 0.632–0.719). Among single-region models, the ITR model ranked second with an AUC of 0.816 (95% CI: 0.772–0.845), while the PTR and PPR models exhibited relatively lower predictive capabilities.

The superior performance of the combined model was further validated in both the validation (*n* = 36) and external test (*n* = 50) sets. In the validation set, the combined model achieved the highest AUC of 0.875 (95% CI: 0.851–0.940), along with remarkable sensitivity (0.872), specificity (0.848), accuracy (0.866), and F1-score (0.725). Similarly, in the external test set, the combined model maintained robust performance, achieving an AUC of 0.825 (95% CI: 0.808–0.852), significantly outperforming single-region models. Across all datasets, the PTR model consistently demonstrated the lowest performance, with AUC values ranging from 0.658 to 0.692, while the PPR model exhibited intermediate performance.

These findings highlight the significant advantage of integrating information from multiple regions (ITR, PTR, and PPR), which enhances the predictive capability of MRI-based models for BCR prediction. The complementary nature of features derived from different prostatic regions is evident, and the consistent performance across external validation underscores the robustness and generalizability of the combined approach.

### 4.3. Performance of the MRI-Based Model, Clinical Model, and Integrated Model

The comparative analysis of three predictive models, as detailed in Table 3, demonstrated that the integrated model consistently outperformed the MRI-based and clinical models across multiple evaluation metrics and datasets. In the training set (*n* = 146), the integrated model achieved the highest performance, with an AUC of 0.861 (95% CI: 0.835–0.872), surpassing both the MRI-based model (AUC: 0.835, 95% CI: 0.812–0.855) and the clinical model (AUC: 0.725, 95% CI: 0.664–0.753). Furthermore, the integrated model exhibited enhanced sensitivity (0.846), specificity (0.868), accuracy (0.855), and F1-score (0.718), outperforming its counterparts in these key metrics.

This performance advantage was sustained in the validation set (*n* = 36), where the integrated model demonstrated robust results with an AUC of 0.857 (95% CI: 0.836–0.894) and a notably high specificity of 0.882 (95% CI: 0.854–0.923). In the external test set (*n* = 50), the integrated model further confirmed its generalizability, maintaining superior performance with an AUC of 0.835 (95% CI: 0.818–0.869), outperforming the MRI-based model (AUC: 0.825) and substantially exceeding the clinical model (AUC: 0.612).

The clinical model, by contrast, demonstrated significantly lower performance metrics, particularly in the external test cohort, where it showed marked deterioration across all parameters (sensitivity: 0.579; specificity: 0.672; accuracy: 0.637; F1-score: 0.351). In comparison, both the MRI-based and integrated models maintained stable performance across all datasets, underscoring their robust generalizability. The consistent superiority of the integrated model across different datasets highlights the value of combining clinical and MRI-based features, which provide complementary information and enhance the reliability and accuracy of BCR prediction in clinical practice.

**Figure 3:**
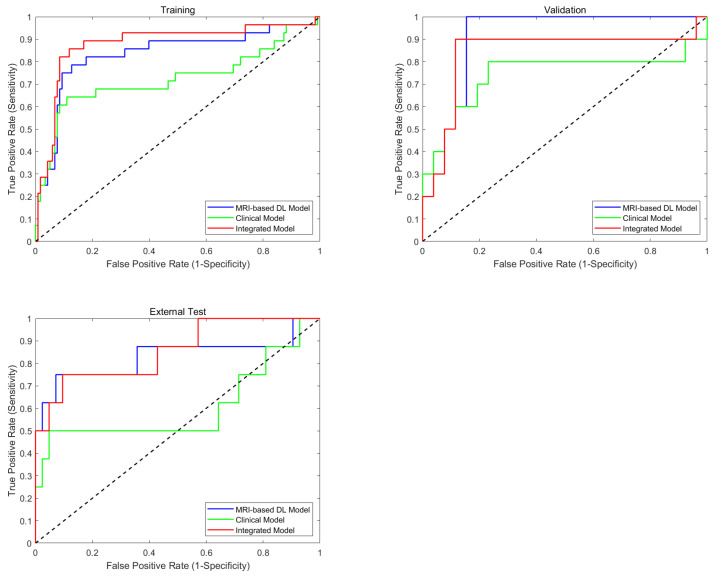
Diagnostic performance of predictive models. Comparison of ROCs of training, validation, and test sets on MRI-based model (combined model), clinical model, and integrated models for BCR prediction.

The ROC curves presented in Figure 3 provide further graphical evidence of the models’ comparative performance across all three datasets. The ROC curve analysis shows that the integrated model (red curve) and multi-region combined model (blue curve) perform best in all three datasets. Using the DeLong test for statistical comparison, the integrated model demonstrates significant advantages over the clinical model in the training set (*p* < 0.001), validation set (*p* = 0.038), and external test set (*p* < 0.001). The performance improvement of the integrated model (with clinical parameters) compared to the MRI-only combined model reached statistical significance in the training set (*p* = 0.043), while the differences were not significant in the validation set (*p* > 0.05) and the external test set (*p* > 0.05). These ROC curves effectively illustrate the consistent superiority of the multi-region multi-sequence MRI feature encoder across all three sets, while also highlighting the limitations of relying solely on clinical parameters for BCR prediction.

## 5. Discussion

T2WI-FS and DWI sequences are essential MRI sequences with distinct characteristics [29]. T2WI-FS [30] enhances the visualization of water-containing tissues while suppressing fat signals, enabling detailed anatomical delineation. Simultaneously, DWI quantifies water-molecule mobility, offering crucial insights into tissue cellularity and potential pathological changes [31]. According to a pivotal study [32], the integration of these sequences has demonstrated superior diagnostic accuracy compared to single-sequence approaches in PCa detection. The standardized acquisition protocols ensure inherent spatial coherence between T2WI-FS and DWI sequences, with matched slice positions and numbers. By leveraging this natural correspondence, we developed a unified CNN-based architecture that processes corresponding T2WI-FS and DWI slices through a shared encoder network. This design enables a simultaneous analysis of structural and functional imaging characteristics while maintaining precise spatial registration between modalities. Our architectural approach offers several key advantages. First, the shared convolutional layers facilitate the learning of cross-modal correlations, capturing both anatomical features from T2WI-FS and corresponding diffusion patterns from DWI. Second, the unified backbone network optimizes computational efficiency by eliminating redundant feature-extraction pathways. Third, the integrated feature fusion in deeper network layers enables the detection of subtle pathological changes that might be overlooked in independent sequence analysis. This comprehensive feature representation, combining both structural and functional information, ultimately enhances the model’s diagnostic capabilities through sophisticated multi-modal learning.

In this study, AUC, sensitivity, specificity, accuracy, and F1-score were applied to evaluate the model’ s prediction performance. AUC is a premier evaluation metric due to its inherent robustness against class imbalance and independence from threshold selection. This makes it particularly valuable for assessing a model’s prediction ability. Unlike other metrics that are measured based on specific thresholds, AUC considers all possible thresholds to comprehensively evaluate a model’s ability to distinguish between positive and negative classes. Among threshold-dependent metrics, sensitivity quantifies the model’s proficiency in correctly identifying actual positive cases (minimizing missed diagnoses), while specificity measures its ability to accurately recognize negative cases (preventing unnecessary treatments). Although accuracy provides a general measure of overall correctness, the F1-score offers a more balanced perspective by combining precision and sensitivity (recall), particularly useful for evaluating prediction performance on imbalanced datasets, as it emphasizes the correctness of positive case predictions. BCR patients are relatively rare in clinical practice, resulting in a class imbalance between recurrence and non-recurrence cohorts. However, accurately identifying these high-risk patients is crucial for clinical decision making and personalized treatment planning. Compared to traditional models that rely solely on clinical indicators, our integrated model—which combines multi-region, multi-sequence MRI features with clinical data—has achieved improvement in prediction accuracy for positive samples (BCR patients). This finding highlights the potential of multimodal data fusion in addressing class imbalance problems.

Previous studies [16,17] have demonstrated the potential of radiomics features extracted from the ITR in predicting postoperative BCR in PCa patients. Although these single-region analyses showed promising results, current molecular pathology research has revealed that tumor progression is significantly influenced by complex interactions between cancer cells and their surrounding microenvironment [33]. To address these limitations and enhance predictive accuracy, we developed a comprehensive multi-region multi-sequence analysis framework incorporating three distinct anatomical regions: ITR, PTR, and PPR. Each region contributes unique biological information critically for understanding disease progression. The ITR captures tumor heterogeneity patterns, the PTR reflects the immediate tumor–host interface where critical molecular interactions occur, and the PPR provides information about the broader tissue environment that may influence tumor behavior. The results indicated that the combined model, which incorporates features from the ITR, PTR, and PPR, outperformed models based on individual image regions. On the external test set, the combined model achieved an AUC of 0.825, compared to 0.803 for the ITR-based model, 0.658 for the PTR-based model, and 0.684 for the PPR-based model. This multi-region approach represents a significant advancement over traditional single-region analyses. By integrating complementary spatial information, our method provides a more comprehensive assessment of the tumor ecosystem, encompassing both intrinsic tumor characteristics and microenvironmental factors. Multiple studies [20] have validated the biological significance of these regions in prostate cancer progression and their collective contribution to BCR prediction. The integration of these spatially distinct but biologically interconnected regions not only enhances our understanding of tumor biology but also provides superior prognostic value compared to conventional single-region approaches.

U-Net [34], with its distinctive encoder–decoder architecture enhanced by skip connections that preserve spatial information for precise boundary delineation, has become the most widespread image-segmentation framework across all medical imaging modalities. Meanwhile, our MRMS-CNNFormer was specifically developed to predict BCR probability in prostate cancer patients by integrating multi-region multi-sequence MRI features with clinical data. Both frameworks include encoders; the CNN-based encoder in MRMS-CNNFormer is specifically optimized for imaging feature extraction. We utilized ResNet18 as the backbone network, effectively mitigating the vanishing gradient problem through residual connections while enhancing deep feature-extraction capabilities. The pretrained weights for ResNet18 on large-scale datasets like ImageNet are readily available, enabling effective transfer learning given our relatively limited dataset size. Similarly, researchers also use pretrained networks as encoders for U-Net to perform image segmentation [35,36]. Importantly, our encoder is designed to simultaneously process dual-channel inputs (T2WI-FS and DWI), learning complementary information through shared convolutional layers, and we introduced an axial spatial information-embedding module that preserves spatial relationships in 3D volumetric data. Moreover, our architecture seamlessly integrates clinical features with imaging features through dimensional expansion, and is then combined with a transformer-based encoder to comprehensively capture the complex characteristics of the prostate cancer microenvironment.

Although graph neural networks can establish multi-region multi-sequence feature fusion, they are constrained by local information passing and require explicit topological graph structure construction. Similarly, while hybrid attention mechanisms combine various attention types, they are limited to predefined receptive fields, struggling to efficiently capture long-range dependencies and requiring complex designs to balance different attention weights. Transformers excel through their self-attention mechanisms that directly establish global feature correlations and simultaneously attend to multiple representational subspaces, allowing for more flexible adaptation to irregular anatomical structures and showing outstanding performance in cross-modal medical data fusion. Abdelhalim et al. [37] developed a 3D multi-branch CNN and vision transformer-integrated architecture for predicting prostate cancer response to hormonal therapy, with branches corresponding to T2-MRI and DW-MRI sequences. Similarly, Dai et al. [25] introduced TransMed, a hybrid CNN–transformer framework that encodes multi-sequence patch images for medical image classification. By contrast, the MRMR-CNNFormer in our current study uniquely integrates multi-region (intratumoral, peritumoral, and periprostatic) and multi-sequence (T2WI-FS and DWI) MRI images with clinical parameters, capturing critical microenvironmental interactions that significantly enhance prostate cancer BCR prediction after radical prostatectomy (with an AUC of 0.835 and an accuracy of 0.819 on the independent external testing cohort (*n* = 50)). Zhong et al. [19] utilized radiomics features extracted using Inception-Resnet v2 network from multiparametric MRI (T1, T2, and DWI sequences) and Adaboost classifier to predict BCR after radiation therapy in localized prostate cancer patients, achieving an AUC of 0.73 and an accuracy of 0.74 in the test cohort (*n* = 18). Wu et al. [38] developed and validated a bi-parametric MRI-based radiomics-clinical combined model to predict BCR after prostate cancer surgery or neoadjuvant therapy, achieving superior performance in the large testing cohort (*n* = 121) with an AUC of 0.841 (95% CI 0.758–0.924). Our MRMS-CNNFormer delivered highly competitive prediction performance relative to other methods, especially model deployment with limited sample size. It should be noted that although transformer-based deep learning approaches usually require large datasets for training, which may be a limitation in medical applications, the usage of data augmentation, architectural design, and regularization techniques can mitigate the issue of limited data [25,39].

The spatial relationships between adjacent axial slices in MRI images are crucial for comprehensive feature extraction [40]. To address the loss of cross-sectional spatial information during 3D-to-2D conversion, we developed an axial spatial information-embedding module and incorporated it into the image features. The module effectively preserves and leverages inter-slice relationships in our model architecture. As for clinical features, they are transformed into high-dimensional representations through 2D expansion, enabling seamless cross-modal fusion with imaging features. This dimensional transformation strategy [41] offers several key advantages: First, it preserves the integrity of clinical information while achieving spatial compatibility with imaging features, avoiding potential information loss from dimensional reduction. Second, it facilitates efficient feature concatenation by maintaining consistent spatial dimensions across modalities, enabling the model to learn complex interactions between clinical and imaging characteristics. Third, this unified representation enhances the model’s capacity to capture complementary patterns across different data types, potentially improving its predictive performance. Additionally, the standardized dimensional space simplifies the computational architecture and reduces the complexity of feature fusion operations. Position embedding plays a vital role in our transformer-based multimodal fusion framework. By encoding spatial information into sequence representations, it preserves crucial anatomical relationships between voxels while enabling the model to maintain spatial context across different modalities [42,43,44]. This mechanism enhances the transformer’s ability to capture both local features and long-range dependencies, facilitating effective cross-modal alignment and spatially coherent feature learning. Additionally, position-aware attention mechanisms [45,46] improve the model’s capacity to integrate complementary information from multiple modalities while preserving anatomical hierarchies essential for medical image analysis.

Our study has several limitations that warrant future investigation. Firstly, despite independent validation with an external dataset yielding promising results, it is constrained by the relatively small sample size. The model’s generalizability across different institutions needs further validation due to variations in MRI equipment and protocols. Although we applied the z-score method for image standardization and batch normalization in the CNN-based encoder, future work will explore more advanced image harmonization techniques [47,48] to enhance the model’s reliability and wider applicability in larger multi-center datasets. Secondly, the current framework lacks the temporal modeling of longitudinal follow-up data. Further exploration will focus on the integration of temporal dynamics and a prospective study to evaluate real-world effectiveness.

## 6. Conclusions

This study developed MRMS-CNNFormer, an innovative multimodal transformer framework that enables the systematic integration of multi-region (intratumoral, peritumoral, and periprostatic regions) and multi-sequence MRI (T2WI-FS and DWI) features with clinical characteristics for predicting BCR in prostate cancer patients. Our framework achieved excellent external testing performance. Specifically, the combined model (AUC = 0.825) significantly outperformed single-region models (AUC = 0.658–0.803), with performance further improved after integrating clinical data (AUC = 0.835). These quantitative results indicate that multi-regional analysis can capture comprehensive information about the tumor microenvironment, while the integration of clinical features with imaging features provides complementary predictive value.

MRMS-CNNFormer provides several important implications for clinical practice. First, the model offers a non-invasive, preoperative BCR risk assessment tool for prostate cancer patients, facilitating individualized treatment decisions. For patients predicted to have high BCR risk, clinicians may consider more aggressive treatment strategies or more frequent follow-up plans. Second, the model highlights the importance of peritumoral tissue in disease progression, supporting the inclusion of tumor microenvironment in clinical assessment. Finally, our results suggest that deep learning methods can extract valuable prognostic information even with limited MRI sequences (T2WI-FS and DWI), which has practical significance for resource-limited healthcare settings.

MRMS-CNNFormer achieves automated prediction via end-to-end training, despite the promising results produced by this advanced deep learning technology, its physical or biological interpretability is limited. Future research will explore the interpretability of the MRMS-CNNFormer framework and further evaluate and validate the model’s effectiveness and reliability on large multicenter datasets.

## Figures and Tables

**Table 1:** Clinical characteristics of patients.

Characteristic	Total (*n* = 232)	Internal Dataset (*n* = 182)	External Dataset (*n* = 50)	*p* Value
Age, years (IQR)	70.0 (65.0, 76.0)	71.0 (65.0, 76.0)	68.0 (62.8, 73.0)	<0.05 ^†^
Gleason score sum, (*n*, %)				<0.05 *
<7	43 (18.5)	37 (20.3)	3 (6.0)	
=7	87 (37.5)	73 (40.1)	16 (32.0)	
>7	102 (44.0)	72 (39.6)	31 (62.0)	
ISUP grade, (*n*, %)				<0.05 *
1	40 (17.2)	37 (20.3)	3 (6.0)	
2	56 (24.1)	47 (25.8)	9 (18.0)	
3	33 (14.2)	26 (14.3)	7 (14.0)	
4	46 (19.8)	35 (19.2)	11 (22.0)	
5	57 (24.6)	37 (20.3)	20 (40.0)	
Clinical stage, (*n*, %)				<0.05 ^‡^
cT2x	190 (81.9)	156 (85.7)	34 (68.0)	
cT3x	42 (18.1)	26 (14.3)	16 (32.0)	
Pathological stage, (*n*, %)				<0.05 ^‡^
pT2	143 (61.6)	103 (56.6)	41 (82.0)	
pT3x	89 (38.4)	79 (43.4)	9 (18.0)	
Perineural invasion (PNI), (*n*, %)				0.193 ^‡^
Positive	125 (53.9)	94 (51.6)	88 (48.4)	
Negative	107 (46.1)	31 (62.0)	19 (38.0)	
Biochemical recurrence (BCR), (*n*, %)				0.443 ^‡^
Yes	46 (19.8)	38 (20.9)	8 (16.0)	
No	186 (80.2)	144 (79.1)	42(84.0)	
CAPRA Score, (*n*, %)				<0.05 ^‡^
≥6	128 (55.2)	93 (51.1)	37 (74.0)	
<6	104 (44.8)	89 (48.9)	13 (26.0)	

Values are presented as *n* (%), or median (interquartile range). *p* values were calculated using Mann–Whitney U test (^†^), Fisher’s exact test (^‡^), and chi-square test (*). *p* values < 0.05 were considered statistically significant.

**Table 2:** Comparison between MRI-based models of multi-region as input in BCR prediction.

	Training (95% CI) (*n* = 146)						Validation (95% CI) (*n* = 36)						External Test (95% CI) (*n* = 50)					
	SENS	SPEC	ACC	F1	AUC	P	SENS	SPEC	ACC	F1	AUC	P	SENS	SPEC	ACC	F1	AUC	P
Intratumoral (ITR)	0.756 (0.727–0.781)	0.844 (0.816–0.865)	0.830 (0.787–0.855)	0.641 (0.596–0.678)	0.816 (0.772–0.845)	**	0.857 (0.836–0.883)	0.814 (0.786–0.845)	0.843 (0.826–0.867)	0.683 (0.647–0.728)	0.858 (0.832–0.874)	*	0.723 (0.701–0.757)	0.818 (0.798–0.843)	0.810 (0.786–0.845)	0.540 (0.508–0.587)	0.803 (0.783–0.822)	**
Peritumoral (PTR)	0.644 (0.618–0.661)	0.685 (0.657–0.713)	0.672 (0.658–0.695)	0.450 (0.420–0.477)	0.664 (0.647–0.708)	***	0.665 (0.642–0.686)	0.723 (0.691–0.754)	0.684 (0.658–0.692)	0.487 (0.458–0.518)	0.692 (0.667–0.732)	***	0.642 (0.627–0.678)	0.682 (0.550–0.724)	0.674 (0.647–0.688)	0.388 (0.314–0.434)	0.658 (0.631–0.692)	***
Periprostatic (PPR)	0.682 (0.657–0.714)	0.744 (0.716–0.768)	0.735 (0.713–0.767)	0.511 (0.477–0.547)	0.699 (0.671–0.737)	***	0.756 (0.730–0.774)	0.699 (0.651–0.720)	0.715 (0.678–0.747)	0.538 (0.495–0.562)	0.731 (0.704–0.762)	***	0.664 (0.647–0.694)	0.741 (0.713–0.758)	0.702 (0.676–0.735)	0.439 (0.410–0.468)	0.684 (0.654–0.723)	***
Combined	0.788 (0.759–0.806)	0.859 (0.834–0.887)	0.852 (0.817–0.866)	0.675 (0.632–0.719)	0.835 (0.812–0.855)	-	0.872 (0.858–0.884)	0.848 (0.825–0.867)	0.866 (0.828–0.897)	0.725 (0.695–0.753)	0.875 (0.851–0.940)	-	0.781 (0.764–0.798)	0.847 (0.819–0.863)	0.819 (0.786–0.855)	0.604 (0.563–0.634)	0.825 (0.808–0.852)	-

Combined: intratumoral, peritumoral, periprostatic regions combined; SENS: sensitivity; SPEC: specificity; ACC: accuracy; AUC: Area Under the Curve. P: ROC curve comparisons between the multi-region combined model and other single-region models; * indicates not significant (*p* > 0.05); ** indicates significant (0.01 < *p* < 0.05); *** indicates very significant (*p* < 0.01).

**Table 3:** Comparison between MRI-based, clinical, and integrated models in BCR prediction.

	Training (95% CI) (*n* = 146)					Validation (95% CI) (*n* = 36)					External Test (95% CI) (*n* = 50)				
	SENS	SPEC	ACC	F1	AUC	SENS	SPEC	ACC	F1	AUC	SENS	SPEC	ACC	F1	AUC
MRI-based model	0.788 (0.759–0.806)	0.859 (0.834–0.887)	0.852 (0.817–0.866)	0.675 (0.632–0.719)	0.835 (0.812–0.855)	0.872 (0.858–0.884)	0.848 (0.825–0.867)	0.866 (0.828–0.897)	0.725 (0.695–0.753)	0.875 (0.851–0.940)	0.781 (0.764–0.798)	0.847 (0.819–0.863)	0.819 (0.786–0.855)	0.604 (0.563–0.634)	0.825 (0.808–0.852)
Clinical model	0.675 (0.624–0.747)	0.776 (0.643–0.813)	0.753 (0.724–0.787)	0.531 (0.415–0.605)	0.725 (0.664–0.753)	0.828 (0.694–0.907)	0.723 (0.684–0.807)	0.758 (0.725–0.798)	0.592 (0.496–0.702)	0.745 (0.721–0.796)	0.579 (0.546–0.633)	0.672 (0.634–0.681)	0.637 (0.611–0.679)	0.351 (0.315–0.383)	0.612 (0.574–0.646)
Integrated model	0.846 (0.781–0.883)	0.868 (0.769–0.903)	0.855 (0.827–0.881)	0.718 (0.584–0.782)	0.861 (0.835–0.872)	0.851 (0.837–0.916)	0.882 (0.854–0.923)	0.867 (0.824–0.883)	0.752 (0.713–0.838)	0.857 (0.836–0.894)	0.774 (0.732–0.819)	0.866 (0.824–0.893)	0.842 (0.815–0.863)	0.625 (0.551–0.688)	0.835 (0.818–0.869)

SENS: sensitivity; SPEC: specificity; ACC: accuracy; AUC: Area Under the Curve.

## Data Availability

The dataset utilized in this study is available from the corresponding author upon request, subject to privacy and ethical restrictions.

## Abbreviations

Abbreviations utilized throughout this manuscript are as follows:

ADC	Apparent Diffusion Coefficient
AUC	Area Under the Curve
BCR	Biochemical Recurrence
CAPRA	Cancer of the Prostate Risk Assessment
CI	Confidence Interval
CNN	Convolutional Neural Network
DCE	Dynamic Contrast-Enhanced
DSC	Dice Similarity Coefficient
DWI	Diffusion-Weighted Imaging
ECE	Extracapsular Extension
ERC	Endorectal Coil
FC	Fully Connected
FOV	Field of View
ITR	Intratumoral Region
MRI	Magnetic Resonance Imaging
mpMRI	multiparametric MRI
PACS	Picture Archiving and Communication System
PCa	Prostate Cancer
PPAC	Pelvic surface Phased Array Coil
PPR	Periprostatic Region
PSA	Prostate Specific Antigen
PTR	Peritumoral Region
ROC	Receiver Operating Characteristic
ROI	Region of Interest
RP	Radical Prostatectomy
SVI	Seminal Vesicle Invasion
T2WI-FS	T2-Weighted Imaging with Fat Suppression
TME	Tumor Microenvironment
